# Hepatic Transcriptome Profiles of Mice with Diet-Induced Nonalcoholic Steatohepatitis Treated with Astaxanthin and Vitamin E

**DOI:** 10.3390/ijms18030593

**Published:** 2017-03-08

**Authors:** Masuko Kobori, Yumiko Takahashi, Mutsumi Sakurai, Yinhua Ni, Guanliang Chen, Mayumi Nagashimada, Shuichi Kaneko, Tsuguhito Ota

**Affiliations:** 1 Food Research Institute, National Agriculture and Food Resrach Organization, Tsukuba, Ibaraki 305-8642, Japan; yumitala@affrc.go.jp (Y.T.); imustum@affrc.go.jp (M.S.); 2 Department of Cell Metabolism and Nutrition, Brain/Liver Interface Medicine Research Center, Kanazawa University, Kanazawa, Ishikawa 920-8640, Japan; shali0145@gmail.com (Y.N.); guanliangc@gmail.com (G.C.); nakanaga@staff.kanazawa-u.ac.jp (M.N.); skaneko@m-kanazawa.jp (S.K.)

**Keywords:** astaxanthin, nonalcoholic steatohepatitis (NASH), comprehensive gene expression analysis, vitamin E, eukaryotic initiation factor-2 (EIF2), peroxisome proliferator-activated receptor α (PPARA)

## Abstract

Astaxanthin alleviates hepatic lipid accumulation and peroxidation, inflammation, and fibrosis in mice with high-cholesterol, high-cholate, and high-fat (CL) diet-induced nonalcoholic steatohepatitis (NASH). It has been proposed as a potential new treatment to inhibit the progression of NASH in humans. In this study, we compared hepatic gene expression profiles after treatment with astaxanthin or the antioxidant vitamin E in mice with CL diet-induced NASH. Comprehensive gene expression analyses of the livers of mice fed a standard, CL, or CL diet containing astaxanthin or vitamin E for 12 weeks were performed using a DNA microarray. Both astaxanthin and vitamin E effectively improved gene expression associated with eukaryotic initiation factor-2 (EIF2) signaling, which is suppressed in NASH by endoplasmic reticulum (ER) stress in the liver. However, astaxanthin did not improve the expression of genes associated with mitochondrial dysfunction. Astaxanthin, but not vitamin E, was predicted to suppress the actions of ligand-dependent nuclear receptors peroxisome proliferator-activated receptors, (PPAR) α (PPARA) and PPARδ (PPARD), and to affect related molecules. Establishing a new therapy using astaxanthin will require elucidation of astaxanthin’s molecular action on the functions of PPARα and related molecules in the livers of mice with diet-induced NASH.

## 1. Introduction

**Figure 1:**
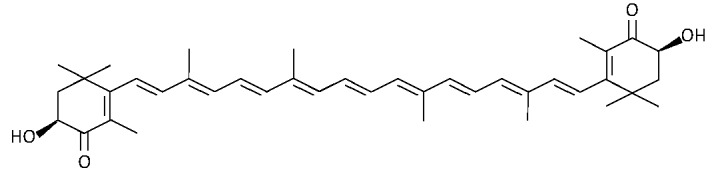
The structure of astaxanthin.

Astaxanthin, widely known as a red pigment in seafood such as salmon, crab, shrimp, and lobsters, is a naturally occurring xanthophyll carotenoid primarily produced by *Haematococcus pluvialis*, other microalgae, and by phytoplankton [1,2]. It is an α-hydroxyketocarotenoid with potent antioxidant activity both in vitro and in vivo [2,3,4,5,6,7], and its radical scavenging activity has been shown to be higher than that of other carotenoids and vitamin E [2,3,4]. Goto et al. suggested that the molecule’s conjugated polyene and terminal moieties trapped radicals in the phospholipid membrane and at the surface (Figure 1) [5]. Astaxanthin from *H. pluvialis* exhibits high bioavailability [2,6,7]. Therefore, although astaxanthin is a non-provitamin A carotenoid, it would be expected to help prevent lifestyle-related diseases by reducing oxidative stress and chronic inflammation [2,7,8,9]. A meta-analysis of randomized controlled trials concluded that supplementation with astaxanthin had a slight plasma glucose-lowering effect [10]. Recently, astaxanthin’s protective effect on liver function has been attracting attention [9,11,12,13]. We have previously shown that astaxanthin ameliorated hepatic steatosis in both ob/ob and high-fat-diet-induced obese mice [14]. It alleviated hepatic lipid accumulation and peroxidation and reversed insulin resistance, hepatic inflammation, and fibrosis in mice with high-cholesterol, high-cholate, and high-fat (CL) diet-induced nonalcoholic steatohepatitis (NASH) [14]. Although vitamin E has been shown to reduce hepatic steatosis and lobster inflammation in patients with NASH, astaxanthin is more effective in preventing and treating NASH in mice [14], suggesting that it improves hepatic steatosis and inhibits the progression of NASH in humans [14]. Thus, astaxanthin may be a promising new treatment for NASH. 

Comprehensive gene expression analysis is a powerful tool for elucidating the properties of natural components and their various physiological functions. We have previously reported that another xanthophyll carotenoid, β-cryptoxanthin, which is found particularly in Satsuma mandarin oranges (*Citrus unshiu* Marc.), attenuated fat accumulation, inflammation, and fibrosis in mice with CL diet-induced NASH [15]. Comprehensive gene expression analysis showed that β-cryptoxanthin effectively suppressed the expression of inflammatory genes but not the expression of the genes associated with steatosis [15]. The study suggested that β-cryptoxanthin suppressed inflammation and the resulting fibrosis primarily by suppressing the increase and activation of macrophages and other immune cells [15]. 

In the present study, to gain a better understanding of the effect of astaxanthin on NASH, we performed comprehensive gene expression analyses of the livers of mice fed a standard, CL, or CL diet containing astaxanthin or vitamin E and compared the gene expression profiles. Our results showed that both astaxanthin and vitamin E improved eukaryotic initiation factor-2 (EIF2) signaling associated with endoplasmic reticulum (ER) stress or global protein synthesis in the livers of mice with diet-induced NASH. We predicted that only astaxanthin would affect the ligand-dependent nuclear receptors peroxisome proliferator-activated receptors (PPARs) and some related factors. Our results revealed the characteristics of the effect of astaxanthin on the livers of mice with diet-induced NASH. 

## 2. Results

### 2.1. Astaxanthin Mainly Improved the Hepatic Gene Expression Associated with Eukaryotic Initiation Factor-2 Signaling in Mice with CL Diet-Induced NASH

**Figure 2:**
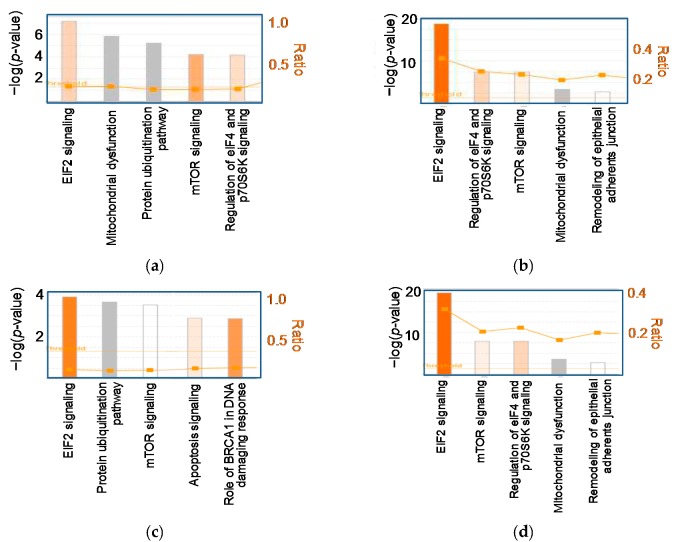
The top five canonical pathways of hepatic genes that were significantly altered by astaxanthin (**a**,**c**); or vitamin E (**b**,**d**) in mice with CL diet-induced nonalcoholic steatohepatitis; (**a**,**b**) Canonical pathways of genes that were significantly up- or downregulated by astaxanthin (*n* = 1137) and vitamin E (*n* = 1397), respectively; (**c**,**d**) Canonical pathways of genes whose expressions were significantly improved by astaxanthin (*n* = 738) and vitamin E (*n* = 1330), respectively. The most significant pathways in the dataset were identified by Ingenuity Pathway Analysis. Orange bars indicate predicted pathway activation. White bars indicate pathways not predicted to be activated or inhibited. Gray bars indicate pathways where no prediction had been made. Orange points connected by lines represent the ratio of the number of genes in a given pathway to the total number of genes in the reference set that make up that pathway. EIF2, eukaryotic initiation factor-2; mTOR, mammalian target of rapamycin.

**Figure 3:**
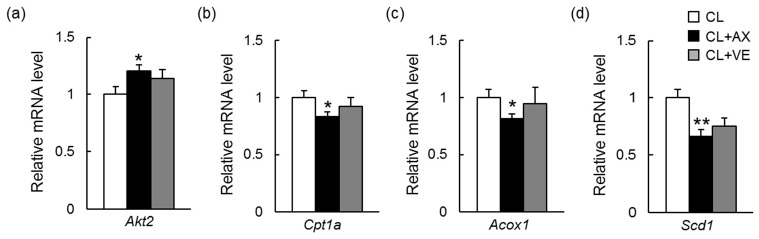
Hepatic mRNA expression of EIF2 signaling-, mitochondrial dysfuction-, peroxisome proliferator-activated receptor α (PPARA)-, and PPARδ (PPARD)-related molecules assessed by quantitative real-time PCR (qPCR). (**a**) EIF2 signaling-related molecule, *Akt2* mRNA expression in the liver of mice with CL diet-induced nonalcoholic steatohepatitis; (**b**) Mitochondrial dysfuction-related molecule, *Cpt1a* mRNA expression in the liver of mice; (**c**) PPARα-related molecule, *Acox1* mRNA expression in the liver of mice; (**d**) PPARδ-related molecule, *Scd1* mRNA expression in the liver of mice. * *p* < 0.05, ** *p* < 0.01 vs. the CL diet.

As we previously reported, supplementation with 0.02% astaxanthin reduced lipid accumulation and peroxidation and attenuated inflammation and fibrosis in the livers of C57BL/6J mice fed a CL diet for 12 weeks [14]. Astaxanthin was more effective than vitamin E in preventing diet-induced NASH [14]. In the present study, therefore, we performed transcriptome analysis using a DNA microarray on the livers of mice fed normal chow (NC), CL diet (CL), CL containing 0.02% astaxanthin (CL + AX), or CL containing vitamin E (CL + VE) for 12 weeks. To elucidate the astaxanthin-affected gene expression profiles associated with NASH, we established which genes were differentially expressed between the NC and CL groups and between the CL and CL + AX groups using ANOVA followed by Welch’s *t*-test (*n* = 5, *p* < 0.05). This showed that 8848 genes were differentially expressed between the NC and CL groups. It is likely that these gene expressions were associated with NASH. Among these genes, 1137 were significantly up- or downregulated by astaxanthin; Table 1a shows the top five biological functions of these genes. Astaxanthin changed the expression of genes associated with cell death and inflammation in the livers of mice with diet-induced NASH. In 738 of these 1137 genes, astaxanthin resulted in expression levels closer to those of the NC group, i.e., the expression was considered to have improved. Figure 2a,c show the top five canonical pathways of the genes whose expressions were significantly altered and significantly improved, respectively, by astaxanthin. Astaxanthin improved the expression of genes associated with the signaling of EIF2, which is required for the initiation of protein translation and is involved in the response to ER stress (Figure 2c and Figure S1). We confirmed that astaxanthin increased mRNA expression of the target molecules of EIF2 signaling, such as *Akt2*, by quantitative real-time PCR (qPCR) (Figure 3). Astaxanthin also improved signaling of the mammalian target of rapamycin (mTOR), which is involved in cell survival and proliferation, and other canonical pathways associated with protein degradation, cell death, and DNA damage, but not the pathway associated with mitochondrial dysfunction (Figure 2a,c and Figure S2). Additionally, qPCR results showed that astaxanthin decreased the mRNA expression of mitochondrial dysfunction related genes such as *Cpt1a* (Figure 3). Vitamin E significantly altered the expression of 1397 genes, expressed differentially between the NC and CL groups, with the expression levels of 1330 of these genes improved to levels closer to those of the NC group. Vitamin E altered the expression of genes associated with lipid metabolism and cell death in the livers of mice with CL diet-induced NASH (Table 1b). As with astaxanthin, vitamin E improved EIF2 and mTOR signaling (Figure 2b,d). However, unlike astaxanthin, it also improved the expression of genes associated with mitochondrial dysfunction (Figure 2b,c and Figure S3).

### 2.2. Astaxanthin Was Predicted to Affect the Ligand-Dependent Nuclear Receptors Peroxisome Proliferator-Activated Receptors δ and α and Retinoid X Receptor α in the Livers of Mice with CL Diet-Induced NASH

**Figure 4:**
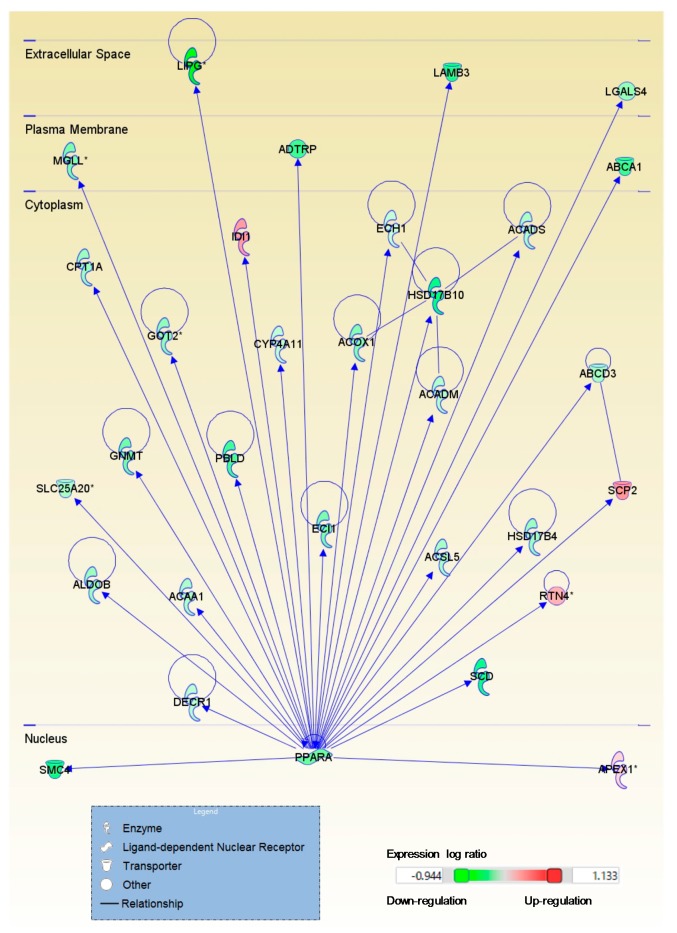
Astaxanthin suppressed the expression of PPARA and the target molecules in mice with nonalcoholic steatohepatitis. →, expression: —, protein-protein binding. *, More than 2 genes were included. The relationship among PPARA, which was predicted to be inhibited by astaxanthin, and the target molecules in the dataset was identified by Ingenuity Pathway Analysis.

**Figure 5:**
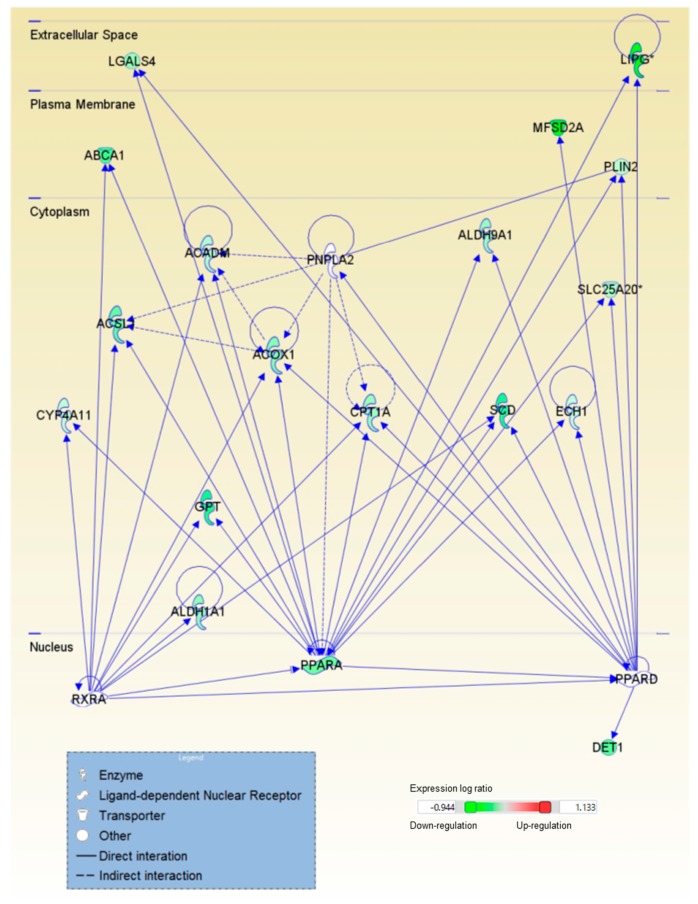
Relationship among PNPLA2, PPARD, PPARA, and RXRA and the target molecules of PNPLA2, PPARD, and RXRA in the dataset regulated by astaxanthin in mice with nonalcoholic steatohepatitis. →, expression: —, protein-protein binding. *, More than 2 genes were included. The network of the molecules was identified by Ingenuity Pathway Analysis.

The upstream regulator analysis of the genes whose expression was altered by astaxanthin or vitamin E indicated that astaxanthin, but not vitamin E, was predicted to inhibit the actions of the ligand-dependent unclear receptors PPARδ (PPARD), PPARα (PPARA), and retinoid X receptor (RXR) α (RXRA) in the livers of mice with diet-induced NASH (Table 2 and Table 3). Figure 4 shows PPARA and the target molecules in the dataset of genes regulated by astaxanthin. Astaxanthin suppressed the expression of the ligand activated transcription factor PPARA and the target genes. Astaxanthin was predicted to inhibit the action of triacylglycerol lipase patatin-like phospholipase domain containing 2 (PNPLA2), which has been shown to activate PPARA activity, and to activate promyelocytic leukemia protein (PML), which has been shown to alter PPAR target genes. The relationship among PNPLA2, PPARD, PPARA, and RXRA and the target molecules were shown in Figure 5. In addition, qPCR results confirmed that astaxanthin, but not vitamin E, decreased mRNA expression for PPARA- and PPARD-related molecules (Figure 3). It has been suggested that both astaxanthin and vitamin E would inhibit the inflammatory cytokine interleukin 6 (IL6), which was predicted to be activated in the livers of mice with NASH (Table 2, Table 3 and Table S1). The mTOR complex 2 component, rapamycin-insensitive companion of mTOR (RICTOR), was predicted to be activated by astaxanthin in the livers of mice with NASH and inhibited by vitamin E (Table 2, Table 3 and Table S1, and Figure S3). ER to nucleus signaling 1 (ERN1) activated by ER stress, Ikaros family zinc finger 1 (IKZF1) related to lymphoid differentiation, and CD28 expressed on T cells were predicted to be activated by astaxanthin (Table 2). Aryl hydrocarbon receptor nuclear translocator (ARNT) and nuclear factor (erythroid-derived 2)-like 2 (NFE2L2) were predicted to be activated in CL diet-induced NASH and inhibited by vitamin E (Table 3 and Table S1).

## 3. Discussion

ER stress has been shown to play an important role in the development of steatosis and the progression of NASH [16]. Increased protein synthesis, lipogenesis, lipid transport, and gluconeogenesis disrupt ER homeostasis and the stress induces steatosis, inflammation, and apoptosis in the liver [16,17]. ER stress activates protein kinase RNA-like ER kinase, phosphorylates EIF2α, and inhibits EIF2α-mediated global protein translation. Pathway analysis showed that EIF2 signaling was inhibited in CL diet-induced NASH and improved by both astaxanthin and vitamin E. ER stress is closely related to oxidative stress in the development and progression of nonalcoholic fatty liver diseases (NAFLDs) and other diseases [18]. Astaxanthin has been reported to reduce ER stress along with a reduction in the activity of nuclear factor-κB (which increases the expression of inflammation genes) in the livers of high-fructose and high-fat diet-fed mice [19]. Astaxanthin is likely to directly or indirectly alleviate ER stress through antioxidant activity. As with vitamin E, astaxanthin significantly reduced the levels of the oxidative stress marker thiobarbituric acid reactive substances (TBARS) in the livers of mice with NASH [14]. Vitamin E has been used as a therapeutic component for NAFLD through its inhibition of reactive oxygen species production [13]. Our results suggested that vitamin E effectively reduced ER stress and improved EIF2 protein translation. 

PPARs regulate lipid and glucose metabolism in the liver. The expression of *Ppara*, which induces genes involved in mitochondrial fatty acid oxidation, is reduced in NAFLD [20]. The expression of *Ppara* negatively correlated with the severity of NASH in patients [21]. The selective PPARD ligand improves hepatic steatosis [20]. PPARD was predicted to be inhibited in the livers of mice with NASH (Supplemental Table S1). PPARA forms heterodimers with RXRA and binds to the PPARA response element of genes [20]. In the present study, astaxanthin but not vitamin E was predicted to inhibit PPARA, PPARD, and RXRA in the livers of mice with NASH. Astaxanthin has been reported to be a PPARA agonist and to reduce lipid accumulation in hepatocytes [22]. Although astaxanthin suppressed, rather than improved, the expression of genes regulated by PPARA, it may directly affect PPARA itself. Further study is required to elucidate the role of astaxanthin on NASH via these ligand-dependent nuclear receptor activities.

The effect of astaxanthin on PPARA activity is likely to disturb the improvement in hepatic gene expression in mice with NASH. Astaxanthin did not improve the expression of genes associated with mitochondrial dysfunction, probably because it reduced mitochondrial function by suppressing the PPARA activity. RICTOR deficiency has been shown to increase the mitochondrial membrane potential [23]. RICTOR may be predicted to be activated by astaxanthin because of the suppression of mitochondrial function and other effects through inhibiting PPARA or PPARD activities (Figure S4). PNPLA2 hydrolyzes triacylglycerol and produces fatty acids and therefore, increases PPARA activity [24]. PML has been shown to control PPAR and to regulate the expression of the PPAR target gene [25]. It has been suggested that the activation of PML suppresses PPARA activity. Thus, it was predicted that PNPLA2 would probably be inhibited and PML would probably be activated by astaxanthin. ERN1, also known as inositol-requiring enzyme 1α, is activated by ER stress and generates functional spliced X-box binding protein 1s (XBP1s) directly binding to activate the promoter of *Ppara* [26]. The phosphorylation and dephosphorylation of EIF2α have been shown to regulate the transcription of XBP1s and target genes [27]. It has been suggested that astaxanthin activates ERN1 in the livers of mice with NASH.

Compared with patients without cirrhosis, the expression of CD28 was reported to be reduced in those with cirrhosis and portal hypertension [28]. CD28 and mitogen-activated protein kinase 8 (MAP3K8) related to TCR/CD28-triggered T cell activation have been predicted to be activated by astaxanthin in the livers of mice with NASH [29]. IKZF1, which regulates leukocyte differentiation, was predicted to be inhibited in NASH and to be activated by astaxanthin [30].

Most of the expression levels of genes altered by vitamin E improved to become closer to those of the control NC group. Vitamin E was predicted to inhibit CL diet-activated RICTOR and IL6. ARNT, which regulates insulin-mediated inhibition on gluconeogenesis and lipogenesis, and NFE2L2, which has been reported to protect against diet-induced NASH in mice, were predicted to be inhibited in the livers of mice with diet-induced NASH and activated by vitamin E [31,32]. Although vitamin E was predicted to activate leptin and Myc proto-oncogene protein (MYC), these molecules were not predicted to be inhibited in the CL group. Leptin shows antisteatotic as well as proinflammatory and profibrogenic actions on NASH [33]. MYC is associated with many cellular events including cell growth, differentiation, and inflammation [34]. The effects of vitamin E on the roles of these molecules in diet-induced NASH should be carefully examined. 

## 4. Materials and Methods 

### 4.1. Animals and Treatments

The mice were treated as previously described. Briefly, 7-week-old C57BL/6J male mice (Charles River Laboratory, Yokohama, Japan) were fed NC (CRF-1, Charles River), CL diet (60% calories from fat, 1.25% cholesterol, and 0.5% sodium cholate), CL diet containing 0.02% astaxanthin (Fuji Chemical Industry, Toyama, Japan), or CL diet containing 0.02% vitamin E for 12 weeks. The mice were maintained on a 12/12-h light/dark cycle and were given free access to food and water. The animal procedures were performed in accordance with the standard set out in the Guidelines for the Care and Use of Laboratory Animals at Kanazawa University, Japan. The study protocols (AP132887) were approved by the Institute for Experimental Animals of Kanazawa University on 5 June 2013.

### 4.2. Comprehensive Gene Expression Analysis 

The liver tissues were collected after 2-h fasting and snap-frozen in liquid nitrogen. Total RNA was isolated from the frozen livers using the GenElute mammalian Total RNA Miniprep kit (Sigma-Aldrich Japan, Tokyo, Japan). We then synthesized fragmented biotin-labeled aRNA from the total RNA of each mouse using the GeneChip 3′ IVT Expression Kit (Affymetrix Japan KK, Tokyo, Japan) and hybridized the labeled aRNA to a GeneChip Mouse Genome 430 2.0 array (Affymetrix). The hybridized probe array was stained using GeneChip Fluidics Station 450 (Affymetrix) and scanned using GeneChip Operation Software version 1.4 (GeneChip Scanner 3000; Affymetrix). The data of twenty microarrays (5 per group) have been deposited in NCBI’s gene expression omnibus (GEO) [35] and are accessible through the GEO Series accession number GSE93819 (http://www.ncbi.nlm.nih.gov/geo/query/acc.cgi?acc=GSE93819).

The DNA microarray data was analyzed using Microarray Suite 5.9 (MAS5; Affymetrix) and Subio platform version 1.19 (Subio Inc., Kagoshima, Japan). Statistical analyses of the genes expressed differentially among three groups (NC, CL, and CL + AX or NC, CL, and CL + VE) and two groups (NC and CL, CL and CL + AX, or CL and CL + VE) were performed using Welch’s one-way ANOVA and Welch’s *t*-test, respectively. The corrected *p* values were considered significant if *p* < 0.05. Ingenuity Pathway Analysis (Ingenuity Systems, www.ingenuity.com) was used to identify the biological functions that were most significant to the extracted data set. A right-tailed Fisher’s exact test was used to calculate a *p*-value denoting the probability that each biological function and canonical pathway for that data set was due to a change in the given parameter alone. An activation *z*-score was calculated as a measure of the activation of biological function, canonical pathway, and the functional or translational activation of upstream regulators. An absolute *z*-score of below 2 (inhibited) or above 2 (activated) was considered significant. 

### 4.3. Quantitative Real-Time PCR 

Total RNA was isolated from frozen liver samples using a GenElute Mammalian Total RNA Miniprep Kit (Sigma-Aldrich). cDNA was synthesized using a High-Capacity cDNA Reverse Transcription Kit (Applied Biosystems, Carlsbad, CA, USA). Quantitative real-time PCR (qPCR) was then performed on a CFX384 machine (Bio-Rad, Hercules, CA, USA) using SYBR Green Master Mix (Applied Biosystems, Carlsbad, CA, USA). The primers used for real-time PCR are shown in Supplemental Table S2. The mRNA expression levels in the groups were normalized to those of CL-fed mice.

## 5. Conclusions

In conclusion, comprehensive gene expression analysis showed that astaxanthin and vitamin E effectively improved hepatic gene expression associated with EIF2 signaling in mice with CL diet-induced NASH. Astaxanthin and vitamin E are likely to alleviate ER stress and improve EIF2 signaling, which initiates global protein translation, in the livers of mice with NASH. Although our previous study showed that astaxanthin was more effective than vitamin E in both preventing and treating NASH, some gene expressions associated with the ligand-dependent nuclear receptors PPARA and PPARD were further suppressed by astaxanthin in the livers of mice with NASH. To establish a therapy for patients with NASH using astaxanthin, it is necessary to elucidate the mechanism of astaxanthin on the functions of PPARA and related molecules in the livers of mice with diet-induced NASH. 

## Figures and Tables

**Table 1:** The top five biological functions of hepatic genes that were significantly altered (**a**) by astaxanthin and (**b**) by vitamin E in mice with high-cholesterol, high-cholate, and high-fat (CL) diet-induced nonalcoholic steatohepatitis ^1^.

(**a**)		
**Biological Functions**	***p*-Value**	**No. of Genes Differentially Expressed**
Cell death and survival (8 functions)	8.98 × 10^−3^–7.90 × 10^−5^	28
Organismal injury and abnormalities (22 functions)	4.58 × 10^−2^–7.90 × 10^−5^	45
Hepatic system development and function (4 functions)	3.73 × 10^−2^–1.20 × 10^−3^	20
Inflammatory response (5 functions)	3.12 × 10^−2^–1.20 × 10^−3^	21
Organ development (4 functions)	3.66 × 10^−2^–1.20 × 10^−3^	22
(**b**)		
**Biological Functions**	***p*-Value**	**No. of Genes Differentially Expressed**
Lipid metabolism (5 functions)	3.88 × 10^−2^–4.15 × 10^−4^	12
Molecular transport (5 functions)	2.51 × 10^−2^–4.15 × 10^−4^	20
Small molecule biochemistry (6 functions)	3.88 × 10^−2^–4.15 × 10^−4^	14
Cell death and survival (8 functions)	3.36 × 10^−2^–9.36 × 10^−4^	29
Organismal injury and abnormalities (23 functions)	3.36 × 10^−2^–1.81 × 10^−3^	7

^1^ The most significant functions in the data set were identified by Ingenuity Pathway Analysis.

**Table 2:** Upstream regulators predicted to be altered by astaxanthin in mice with nonalcoholic steatohepatitis ^1^.

Upstream Regulator ^2^	Molecule Type	Predicted Activation State	Activation *z*-score	*p*-Value for the Overlap	Target Molecules in Dataset
PNPLA2	Enzyme	Inhibited	−2.2	4.55 × 10^−4^	ACADM, ACOX1, ACSL1, CPT1A, PPARA
PPARD	Ligand-dependent nuclear receptor	Inhibited	−2.975	1.46 × 10^−1^	ALDH9A1, DET1, ECH1, LGALS4 LIPG, MFSD2A, PLIN2, SCD, SLC25A20
PPARA	Ligand-dependent nuclear receptor	Inhibited	−2.351	8.54 × 10^−3^	ABCA1, ABCD3, ACAA1, ACADM, ACADS, ACOX1, ACSL5, ADTRP, ALDOB, APEX1, CPT1A, CYP4A11, DECR1, ECH1, ECI1, GNMT, GOT2, HSD17B10, HSD17B4, IDI1, LAMB3, LGALS4, LIPG, MGLL, PBLD, RTN4, SCD, SCP2, SLC25A20, SMC4
RXRA	Ligand-dependent nuclear receptor	Inhibited	−2.156	1.37 × 10^−2^	ABCA1, ACADM, ACOX1, ALDH1A1, CYP4A11, GPT
IL6	Cytokine	Inhibited	−2.362	1.00 × 10^0^	ACOX1, IL6ST, MAF, NR3C1, RORA, SMAD7
RICTOR	Other	Activated	2.846	1.83 × 10^−8^	ATP5D, ATP5G2, ATP5O, ATP6V0C, ATP6V1D, COX5A, Cox5b, COX7A2, MCL1, NDUFA2, NDUFA6, NDUFA7, NDUFAB1, NDUFB3, NDUFC1, NDUFC2, Ndufs5, PSMB1, PSMB2, PSMB6, PSMB7, PSMD11, PSMD12, PSMD7, RPL10A, RPL13A, RPL18, RPL23, RPL6, RPL9, Rplp1(includes others), RPS11, RPS15, RPS18, RPS24, RPS26, RPS3, RPS8, RPS9, UQCRHL
ERN1	Kinase	Activated	2.345	3.85 × 10^−2^	DGAT2, FITM2, GPT, PLIN2, SCD, SEC61A1, SRPRA, SURF4, WFS1
PML	Transcription regulator	Activated	2.449	9.73 × 10^−3^	ACADM, ACADS, ACOX1, CPT1A, SCD, SLC25A20
MAP3K8	Kinase	Activated	2.213	1.00 × 10^0^	BMP1, CLIC5, FAAP24, FAM107B, FLNB, IFNGR1, IGF1R
IKZF1	Transcription regulator	Activated	2	1.00 × 10^0^	HNRNPLL, MYO1B, SH3BP5, SULF2
CD28	Transmembrane receptor	Activated	2.433	3.61 × 10^−1^	ATF2, CASP6, CASP8, IFNGR1, IGF1R, MAF, RORA

^1^ The most significant upstream regulators in the data set were identified by Ingenuity Pathway Analysis; ^2^ For explanations of the abbreviations, please see the Abbreviation List.

**Table 3:** Upstream regulators predicted to be altered by vitamin E in nonalcoholic steatohepatitis mice ^1^.

Upstream Regulator ^2^	Molecule Type	Predicted Activation State	Activation *z*-score	*p*-Value for the Overlap	Target Molecules in Dataset
RICTOR	Other	Inhibited	−6.505	1.63 × 10^−11^	Atp5e, ATP6V0A2, ATP6V1A, ATP6V1D, COX4I1, Cox5b, COX6A1, COX6B1, COX7A2, COX7A2L, CYC1, FAU, NDUFA2, NDUFA3, NDUFB7, NDUFC1, NDUFS6, NDUFV1, PSMB3, PSMC2, PSMD13, RPL13A, RPL14, RPL17, RPL18, RPL22, RPL23, Rpl23a, RPL26, RPL28, RPL30, Rpl34 (includes others), RPL35A, RPL38, RPL41, RPL8, RPL9, Rplp1 (includes others), RPLP2, RPS10, RPS11, RPS13, RPS15, RPS21, RPS24, RPS27A, RPS29, RPS6, RPSA, SHFM1
IL6	Cytokine	Inhibited	−2.234	5.03 × 10^−1^	ABCC3, ACOX1, C3, CCR5, F3, IL6ST, MAF, NR3C1, RORA
LEP	Growth factor	Activated	2.543	2.10 × 10^−1^	ABCC3, ACADVL, ACOX1, CYR61, ECH1, FAAH, GAPDH, IL1B, OPLAH, PRDX1, SCD, SOD1
ARNT	Transcription regulator	Activated	2.236	3.62 × 10^−2^	CCR5, ENO1, GAPDH, PGK1, TPI1
NFE2L2	Transcription regulator	Activated	4.498	2.61 × 10^−6^	ABCC3, AKR1A1, AKR7A2, ARF1, ATF7, ATP1A1, CCT3, CDC34, CLPP, COQ7, DDX39B, EIF3C, EIF3G, EPHX1, F10, FTL, GNA11, GSPT1, GSTM5, HACD3, HAX1, HM13, IL1B, MCFD2, MORF4L2, NCKAP1, NFE2L1, PPIB, PRDX1, PSMB3, PSMD13, RAN, RPL18, RPS16, S100A13, SERINC3, SLCO1B3, TPI1
MYC	Transcription regulator	Activated	2.76	9.34 × 10^−2^	ENO1, GAPDH, GPI, HNRNPAB, HNRNPD, KAT2A, NCL, PA2G4, PCK1, PGK1, TPI1, ZFP36L1

^1^ The most significant upstream regulators in the data set were identified by Ingenuity Pathway Analysis; ^2^ For explanations of the abbreviations, please see the Abbreviation List.

## Supplementary Materials

Supplementary materials can be found at www.mdpi.com/1422-0067/18/3/593/s1.

## Abbreviations

NASH	nonalcoholic steatohepatitis
EIF2	eukaryotic initiation factor-2
ER	endoplasmic reticulum
PPAR	peroxisome proliferator-activated receptor
NC	normal chow
CL	CL diet
CL + AX	CL containing 0.02% astaxanthin
CL + VE	CL containing 0.02% vitamin E
mTOR	mammalian target of rapamycin
RXR	retinoid X receptor
PNPLA2	patatin-like phospholipase domain containing 2
PML	promyelocytic leukemia protein
IL6	interleukin 6
RICTOR	rapamycin-insensitive companion of mTOR
IKZF1	Ikaros family zinc finger 1
ERN1	endoplasmic reticulum to nucleus signaling 1
ARNT	aryl hydrocarbon receptor nuclear translocator
NFE2L2	nuclear factor (erythroid-derived 2)-like 2
NAFLD	nonalcoholic fatty liver disease
XBP1s	X-box binding protein 1s
MAP3K8	mitogen-activated protein kinase 8
