# Supplementary material for: Hepatic Transcriptome Profiles of Mice with Diet-Induced Nonalcoholic Steatohepatitis Treated with Astaxanthin and Vitamin E
Source: Int J Mol Sci. 2017 Mar 8;18(3):593. doi: 10.3390/ijms18030593 (PMC5372609; doi:10.3390/ijms18030593)
Supplement: Supplementary file 1 [file ijms-18-00593-s001.zip › ijms-177044-supp-final.pdf]

Supplementary Materials: Hepatic Transcriptome Profiles of Mice with Diet-Induced Nonalcoholic Steatohepatitis Treated with Astaxanthin and Vitamin E

Masuko Kobori, Yumiko Takahashi, Mutsumi Sakurai, Yinhua Ni, Guanliang Chen, Mayumi Nagashimada, Shuichi Kaneko and Tsuguhito Ota

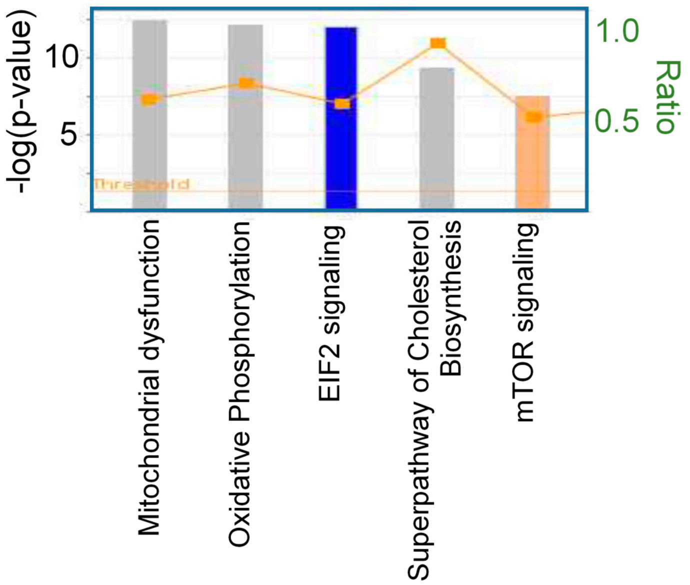

Figure S1. The top five canonical pathways of hepatic genes that were significantly altered in mice with diet-induced nonalcoholic steatohepatitis. Genes that were significantly up- or downregulated in the livers of the CL diet group compared with those in the normal chow group were analyzed by Ingenuity Pathway Analysis to identify the most significant pathways in the data set. The orange bar indicates the predicted pathway activation. The blue bar indicates the predicted pathway inhibition. Gray bars indicate pathways where no prediction had been made. Orange points connected by lines represent the ratio of the number of genes in a given pathway to the total number of genes in the reference set that make up that pathway. EIF2, eukaryotic initiation factor-2; mTOR, mammalian target of rapamycin.

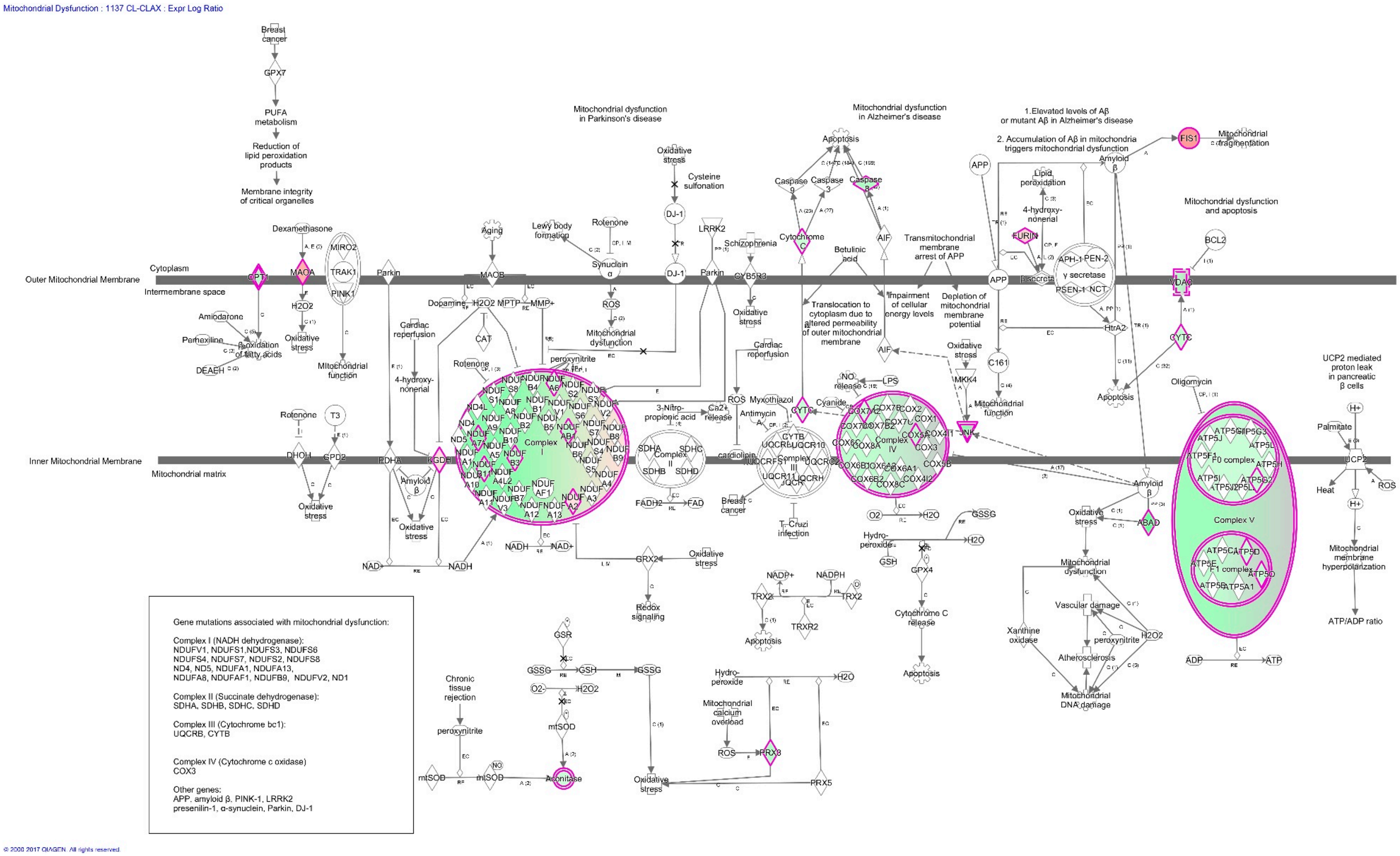

Figure S2. Astaxanthin suppressed the gene expression associated with mitochondrial dysfunction. The Ingenuity Pathway Analysis (IPA) mitochondrial dysfunction pathway of hepatic genes that were significantly altered by astaxanthin.

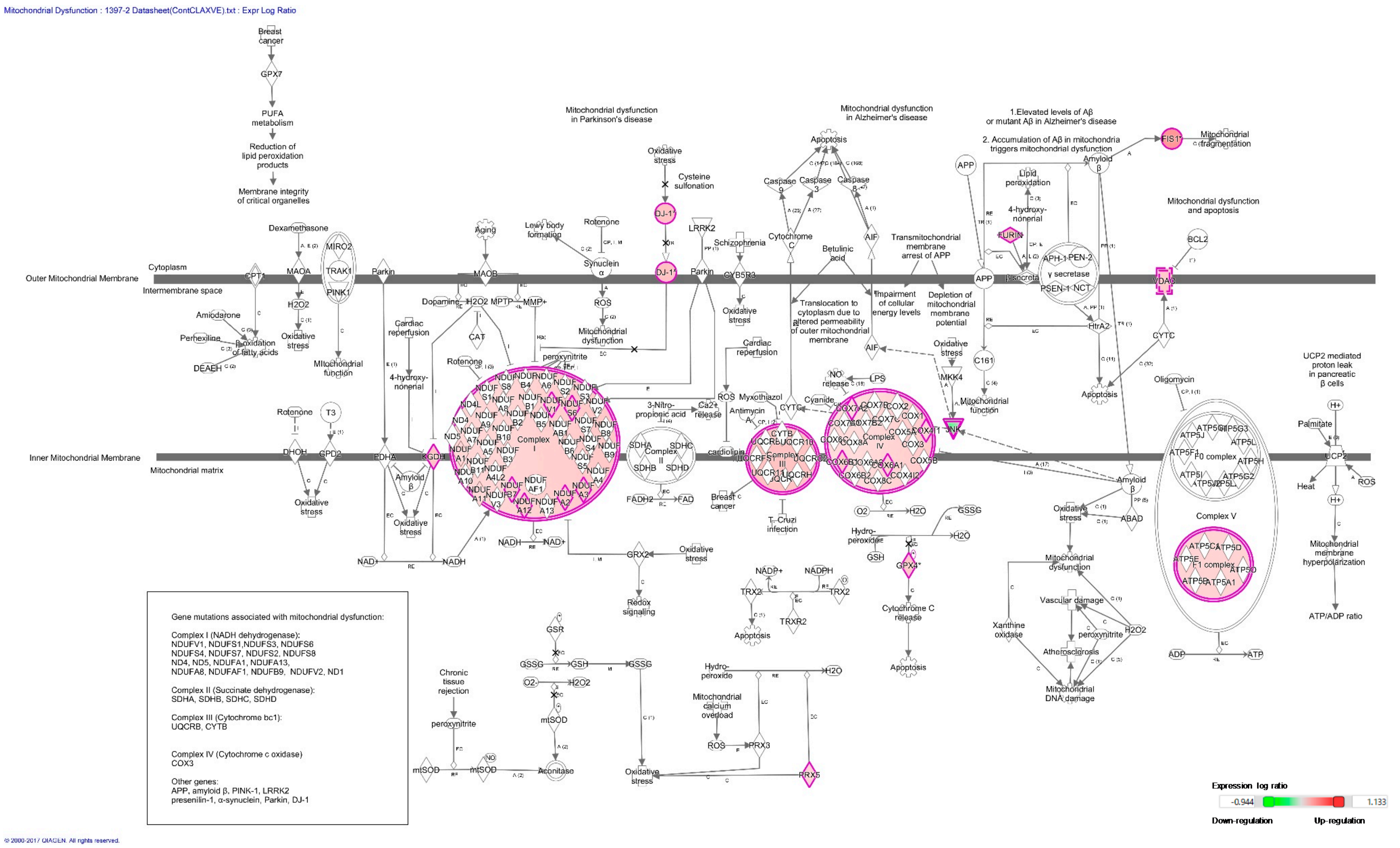

Figure S3. Vitamin E improved the gene expression associated with mitochondrial dysfunction. The IPA mitochondrial dysfunction pathway of hepatic genes that were significantly altered by vitamin E.

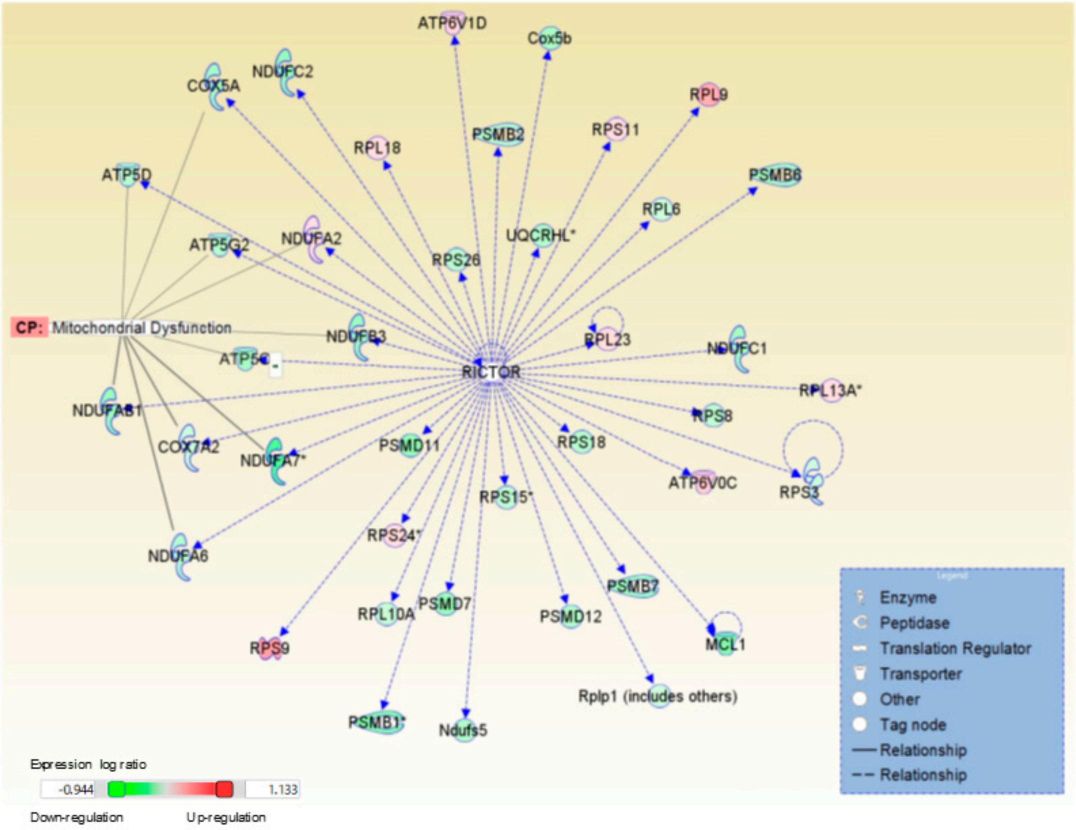

Figure S4. Relationship between rapamycin-insensitive companion of mTOR (RICTOR) and the target molecules in the dataset regulated by astaxanthin in mice with nonalcoholic steatohepatitis. →, expression. The network of the molecules was identified by Ingenuity Pathway Analysis. Most of the genes in the figure are known to be downregulated by RICTOR activation.

Table S2. Primer sequence used for real-time PCR.

Gene	5′ Primer	3′ Primer
Scd1	CAT CAT TCT CAT GGT CCT GCT	CCC AGT CGT ACA CGT CAT TTT
Cpt1a	AAA CCC ACC AGG CTA CAG TG	TCC TTG TAA TGT GCG AGC TG
Acox1	TTA TGC GCA GAC AGA GAT GG	AGG CAT GTA ACC CGT AGC AC
Akt2	CAT AGA TTC TTC CTC AGC ATC AAC	GCT GGT CCA GTT CCA GCG GG
